# Supplementary material for: Automated classification of giant virus genomes using a random forest model built on trademark protein families
Source: Npj Viruses. 2024 Mar 8;2:9. doi: 10.1038/s44298-024-00021-9 (PMC11721082; doi:10.1038/s44298-024-00021-9)
Supplement: Supplementary file 1 — Supplemental Figures [file 44298_2024_21_MOESM1_ESM.pdf]

Supplemental Figures

Automated classification of giant virus genomes using a random forest model built on trademark protein families

Figure S1. Distinct protein family profiles in *Nucleocytoviricota* families. The y-axis denotes the family assignments of giant virus representative genomes, color-coded by family. The x-axis shows different GVOGs. The Not_GV group includes *Mirusviricota* and jumbo phage genomes.

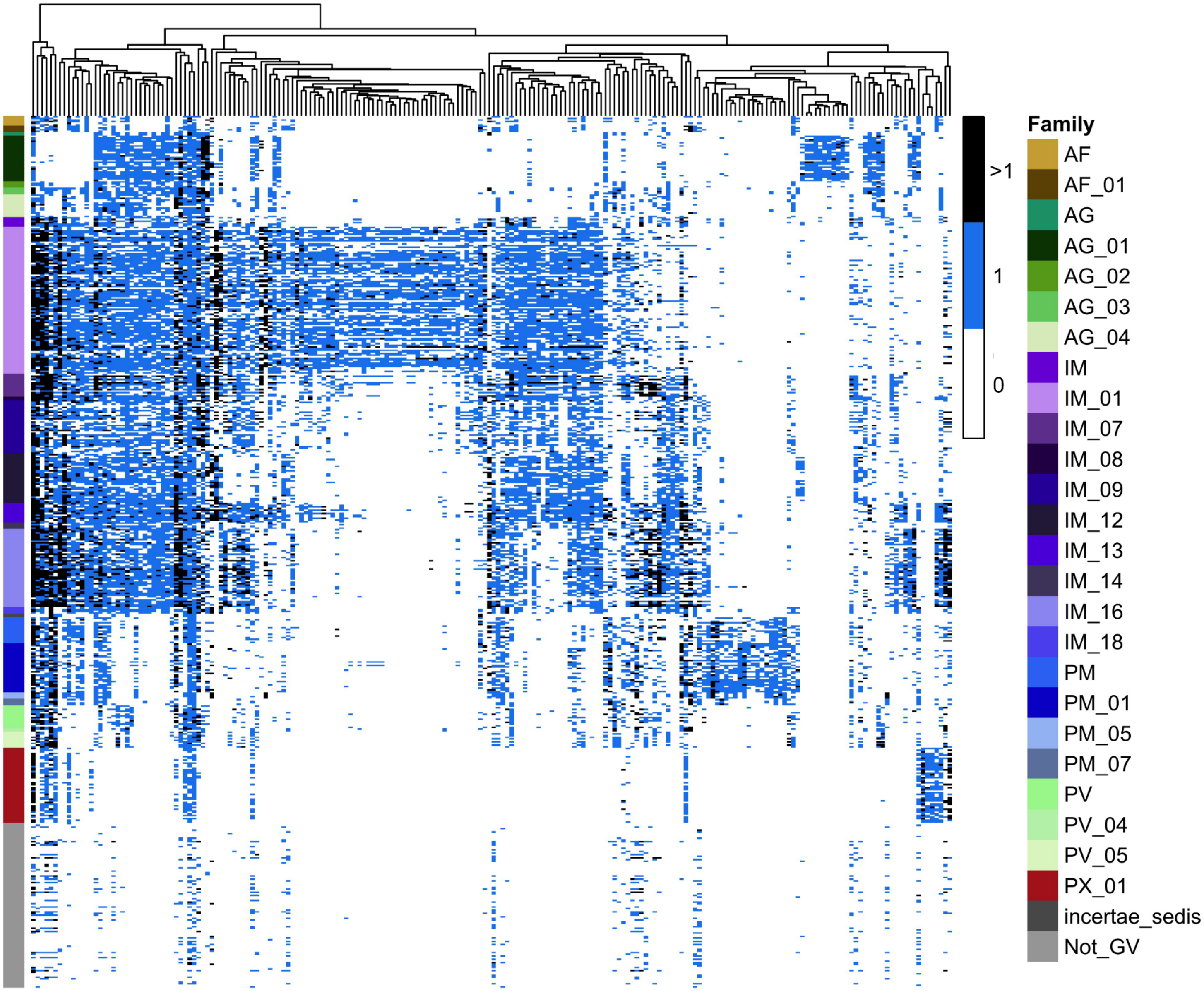

Figure S2. Heatmap of correlation among GVOG features within the initial 625 GVOG set. Colors represent the Spearman correlation coefficients between GVOG features. GVOGs were ordered according to the leaf arrangement in the hierarchical clustering dendrogram at the top. (Top) Dendrogram showing hierarchical clustering of GVOGs based on their distance (derived from Spearman rank-order correlations). Hierarchical clustering was performed using Ward's linkage. The y-axis corresponds to the distance between clusters.

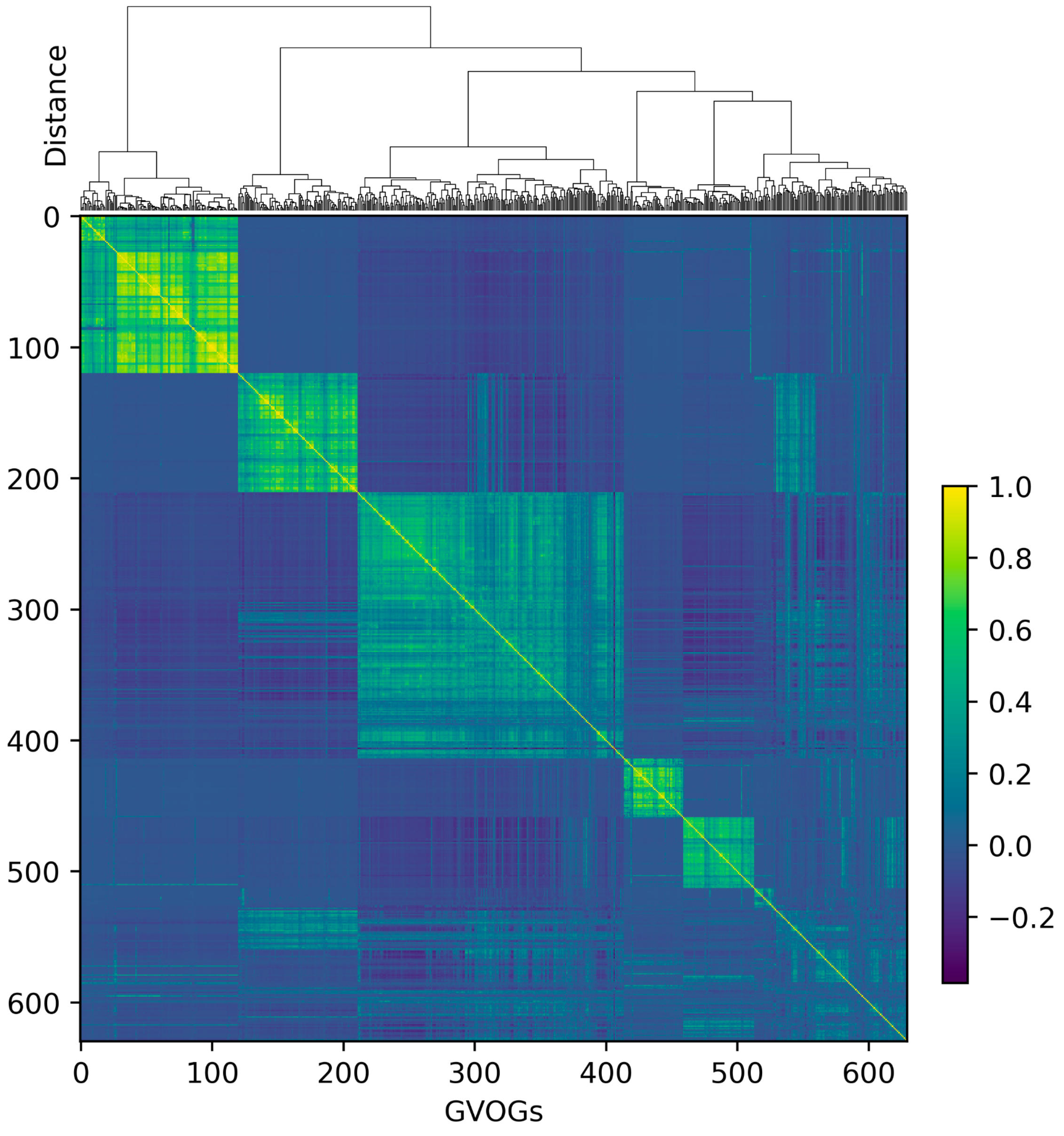

Figure S3. MDI-based feature importances for the 15 most important features in the classification model at the order level (top) and family level (bottom).

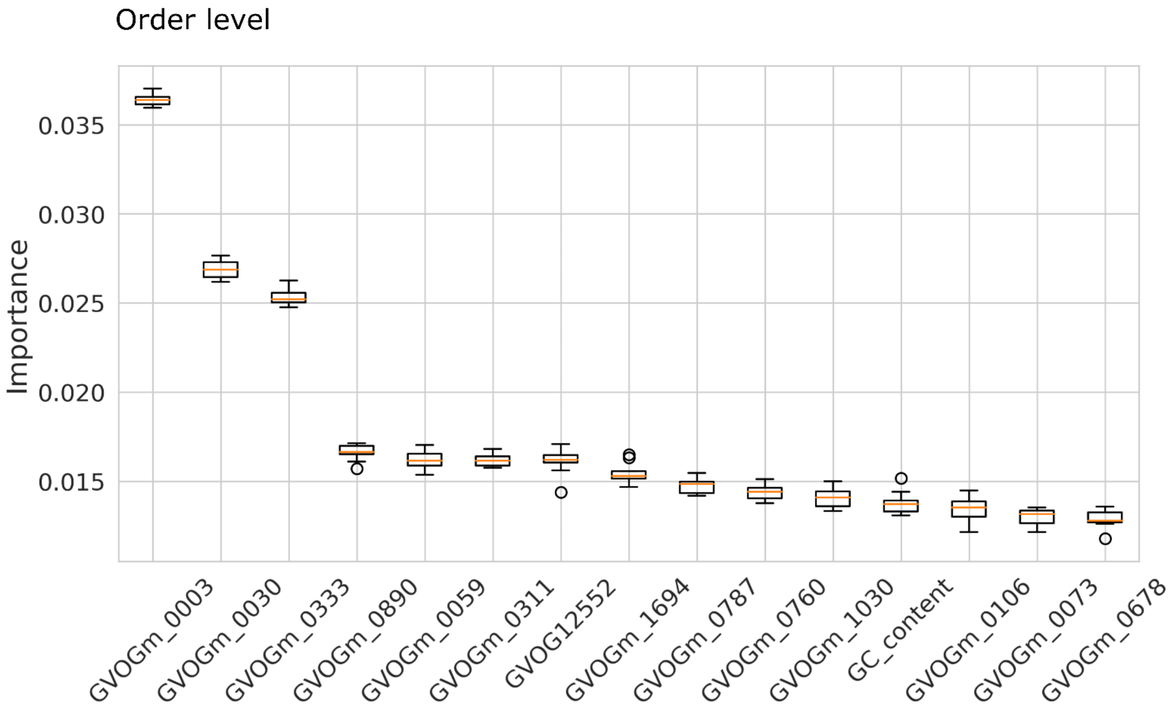

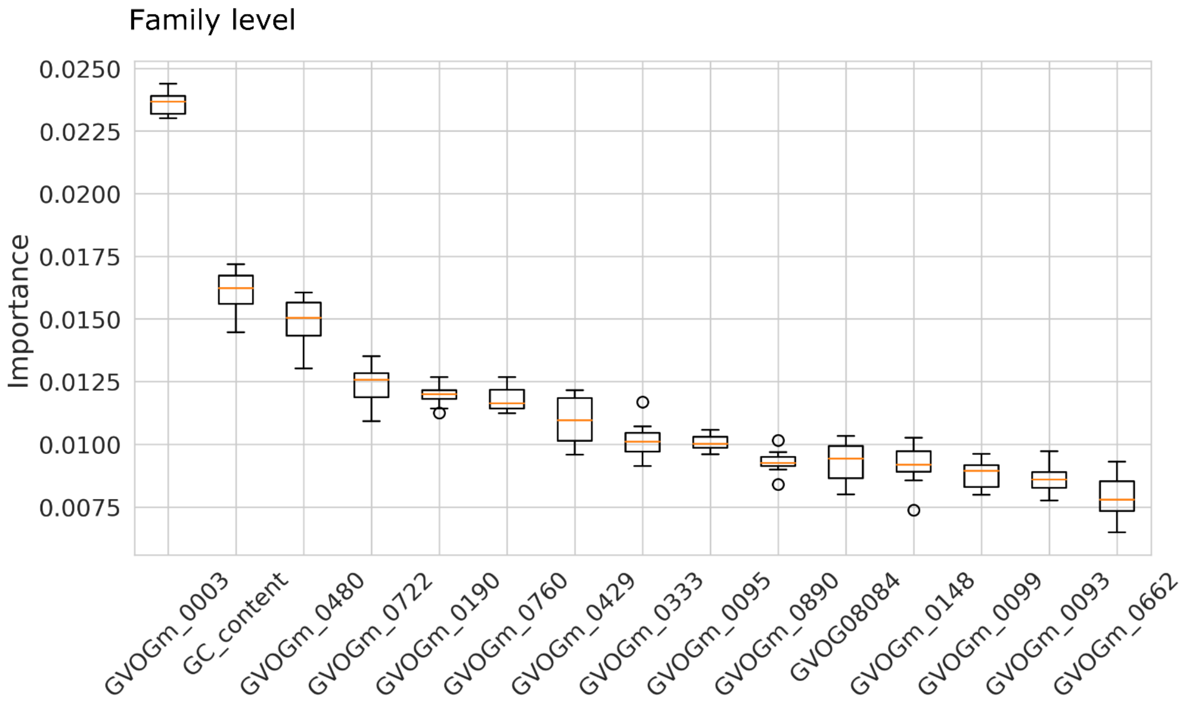

Figure S4. Performance of models feature sets selected by two feature importance mechanisms. Model performance was estimated using 10-fold nested cross-validation.

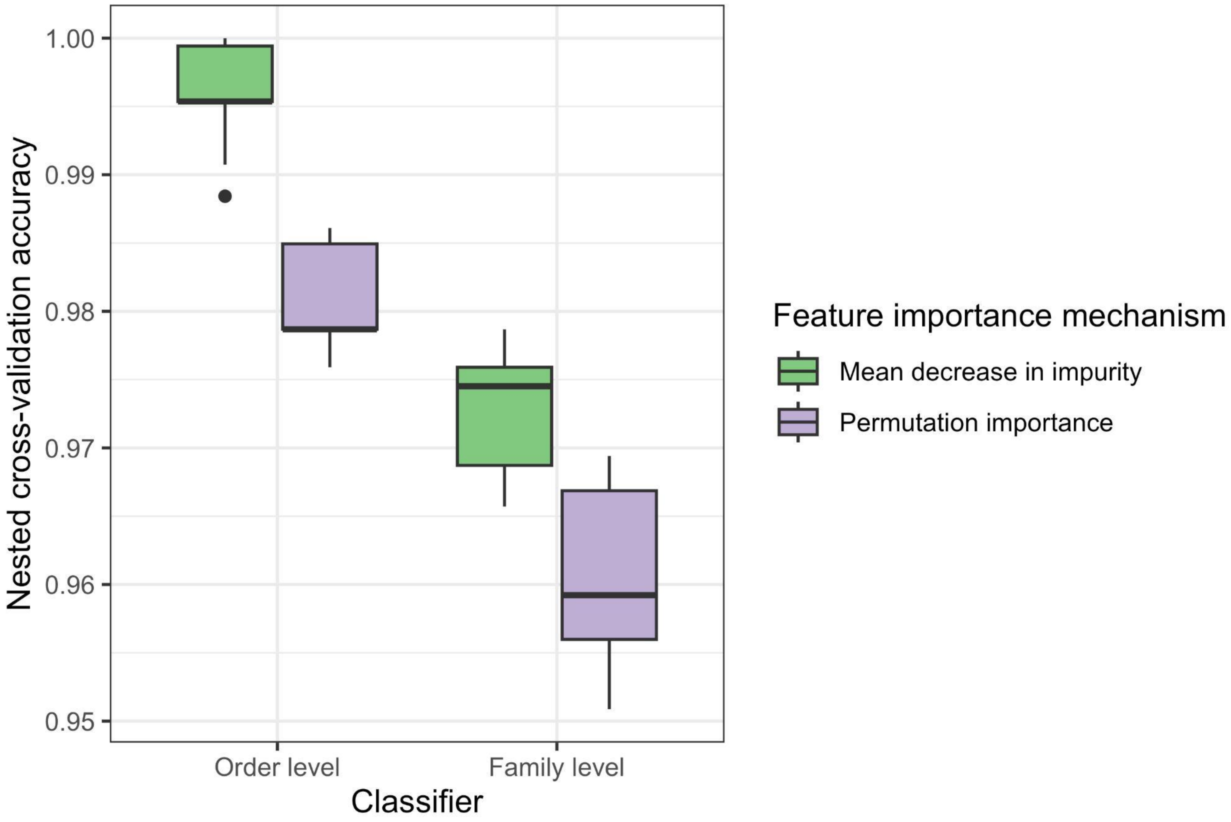

Figure S5. Training sequences from the orders *Algavirales* and *Imitervirales*, visualized in dimension of two t-distributed stochastic neighbour embedding (T-SNE) components. Data dimensions were reduced using PCA and T-SNE. Dots are color-coded based on the major families within the orders *Algavirales* and *Imitervirales*.

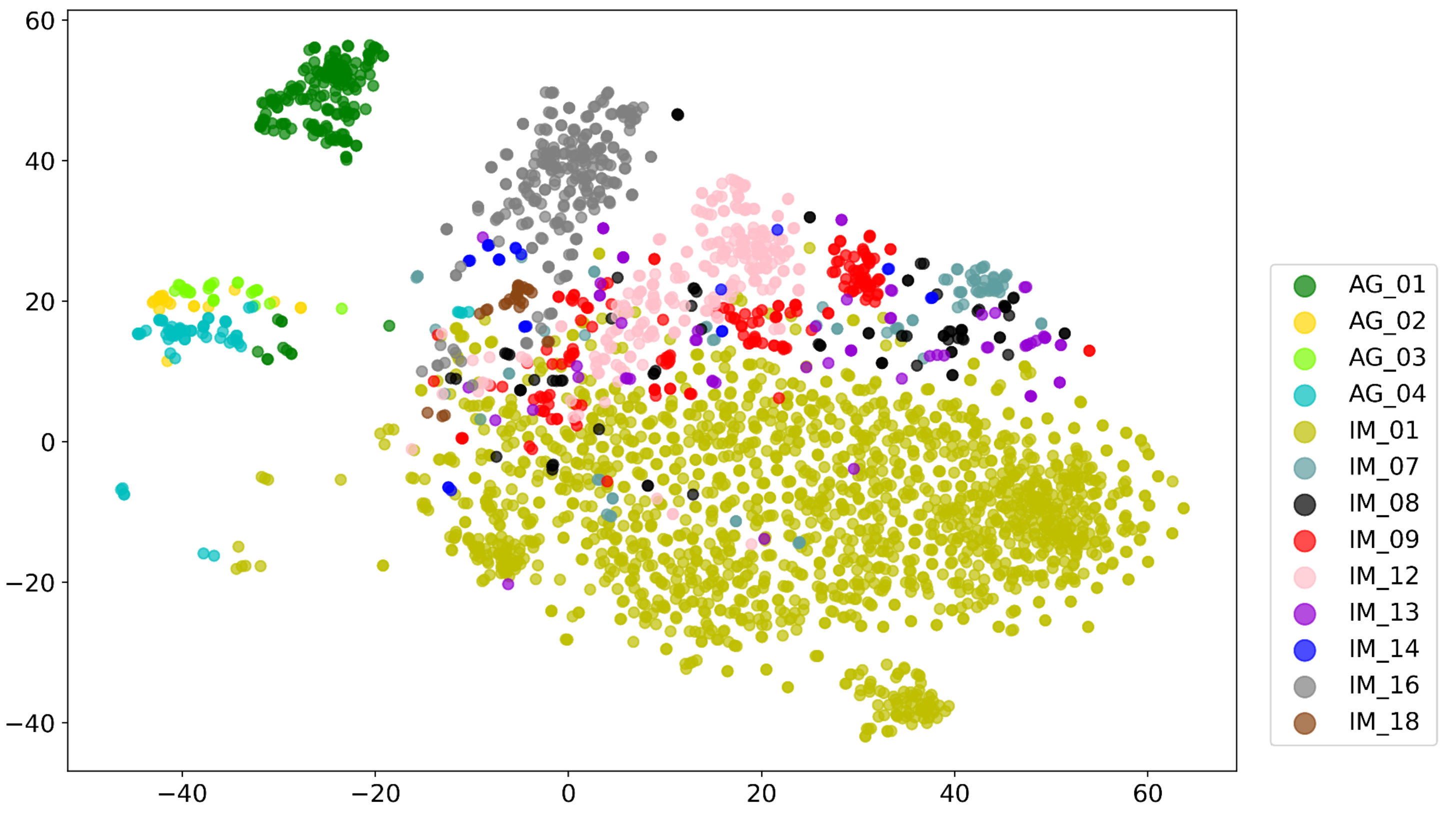

Supplemental Data S1. GVOG features used for classification models at the order and family levels. Features were selected using RF's MDI-based importance.

Supplemental Data S2. Summary of viral genomes included in model training and independent testing.

Supplemental Data S3. Classification report at the family level.

Supplemental Data S4. Summary of reference giant virus genomes included in the custom AAI database.
